# E3 ubiquitin ligase Pirh2 enhances tumorigenic properties of human non-small cell lung carcinoma cells

**DOI:** 10.18632/genesandcancer.123

**Published:** 2016-11

**Authors:** Alexandra Daks, Alexey Petukhov, Olga Fedorova, Oleg Shuvalov, Valeriy Merkulov, Elena Vasileva, Alexey Antonov, Nikolai A. Barlev

**Affiliations:** ^1^ Institute of Cytology, Russian Academy of Sciences, St Petersburg, Russia; ^2^ Almazov Federal North-West Medical Research Centre, Institute of Hematology, St Petersburg, Russia; ^3^ National Research University of Information Technologies, Mechanics and Optics, St Petersburg, Russia; ^4^ MRC Toxicology Unit, Leicester, UK

**Keywords:** Pirh2, *RCHY1*, H1299, non-small cell lung cancer, drug resistance, EMT, xCELLigence

## Abstract

The product of *RCHY1* human gene, Pirh2, is a RING-finger containing E3 ligase that modifies p53 with ubiquitin residues resulting in its subsequent degradation in proteasomes. Transcription of *RCHY1* is regulated by p53 itself thus forming a negative regulatory feedback loop. Functionally, by eliminating p53, Pirh2 facilitates tumorigenesis. However, the role of Pirh2 in cancer cells lacking p53 is yet not well understood. Therefore, we decided to elucidate the role of Pirh2 in p53-negative human non-small cell lung carcinoma cells, H1299. We found that ectopic expression of Pirh2 enhanced cell proliferation, resistance to doxorubicin, and increased migration potential. Ablation of Pirh2 by specific shRNA reversed these phenotypes. Mechanistically, Pirh2 increased mRNA and protein levels of the c-Myc oncogene. The bioinformatics data indicate that co-expression of both c-Myc and Pirh2 strongly correlated with poor survival of lung cancer patients. Collectively, our results suggest that Pirh2 can be considered as a potential pharmacological target for developing anticancer therapies to treat p53-negative cancers.

## INTRODUCTION

Pirh2 was originally identified as an androgen receptor (AR) N-terminal-interacting protein (ARNIP) that exhibited ubiquitin ligase activity [1]. Later, Pirh2, in addition to Mdm2, was found to be one of the principal ubiquitin ligases that targets the major tumor suppressor p53 [2, 3].

P53 exerts its tumor suppressor functions as a transcription factor regulating expression of both coding and non-coding genes whose products induce cell cycle arrest and apoptosis in response to various forms of cellular stress [4, 5]. Under normal conditions p53 is a short-lived protein, which undergoes rapid ubiquitin-dependent degradation in proteasomes [6]. However, upon genotoxic stress p53 becomes post-translationally modified by several covalent moieties, including phosphorylation, acetylation and methylation [7, 8]. Importantly, in response to DNA damage proteasomes themselves undergo covalent modifications that inactivate their proteolytic activity [9] resulting in further stabilization of p53. It was shown that Pirh2, in contrast to Mdm2, was able to ubiquitinate p53 phosphorylated on Ser15, one of the hallmark of p53 activation in response to DNA damage [10]. Thus, Pirh2 seems to be the major regulator of p53 under stress conditions [3]. Yet, p53 activates expression of the Pirh2-encoding *RCHY1* gene, thus forming a negative regulatory feedback loop [11-13]. Besides p53 and its homologs p63 and p73 [14-16], there are several other targets of Pirh2 that play roles in cell cycle regulation, apoptosis activation, DNA-damage response and tumor transformation, such as Chk2, p27^Kip1^ and Polη [17-19]. Pirh2 ubiquitinates these proteins and directs them into the degradation pathway thus affecting apoptosis induction, cell cycle regulation and DNA repair. However the involvement of Pirh2 in these processes still needs further investigation.

Despite the negative effect on p53, the role of Pirh2 in cancer progression is rather obscure. For example, Duan et al. carried out the analysis of Pirh2 expression in human lung neoplasms paired with normal lung tissues. As the result, it was shown that expression of Pirh2 was increased in 27 (84%) of 32 human specimens [20]. Similar results were obtained for Pirh2 expression in prostate cancer. Overexpression of Pirh2 was detected in 73 of 82 (89%) resected human prostate cancer specimens [21]. Overexpression of Pirh2 in hepatocellular carcinoma (HCC) cells was found to correlate with vein invasion, TNM stage and number of tumor nodes [22]. Shimada and colleagues reported that in about 60% cases of human HNSCC increased Pirh2 levels were observed in comparison with 0% of normal mucosa [23]. These data strongly suggest that Pirh2 is an oncogene. On the other hand, genome-wide microarray studies showed that lower levels of Pirh2 mRNA were associated with reduced survival of patients with breast and ovarian cancer, and lung squamous carcinomas [24]. Thus, the role of Pirh2 in tumorigenesis seems to be ambiguous and needs further investigation.

To elucidate the p53-independent role of Pirh2 in lung cancer we examined the effect of Pirh2 on proliferation, invasion potential and drug resistance of H1299 p53-negative lung carcinoma cells.

## RESULTS

### Pirh2 affects proliferation of H1299 cells

To elucidate the role of Pirh2 in p53-negative cancer cells we decided to assess the effect of Pirh2 expression on classical characteristics of tumorigenecity: proliferation, invasion potential, and resistance to anti-cancer drugs. We chose H1299 cells since these lung carcinoma cells are negative for p53 and express relatively low levels of Pirh2 thus making these cells a convenient system to study effects of Pirh2 ectopic expression.

**Figure 1:**
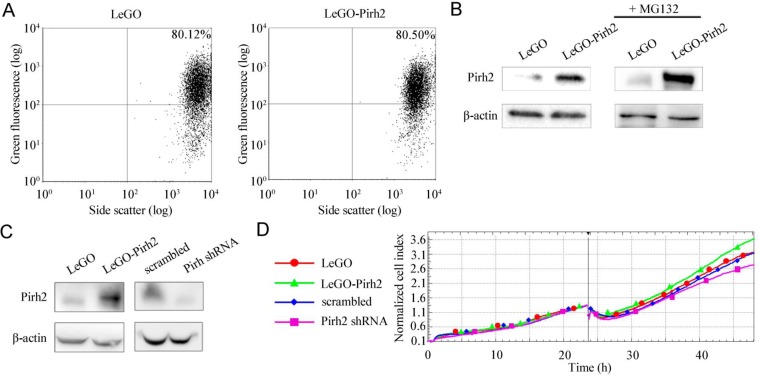
Pirh2 affects proliferation of H1299 cells (A) Evaluation of transduction efficiency of H1299 cells with LeGO- and LeGO-Pirh2 by FACs analysis of GFP-positive cells. (B) Western blot analysis of Pirh2 protein levels in H1299 cells stably expressing LeGO-Pirh2 and LeGO control before (left panel) and after (right panel) the 16 h treatment with 5 μM proteasome inhibitor MG132. (C) Western blot analysis of Pirh2 protein levels in H1299 cells stably expressing LeGO-Pirh2, LeGO, Pirh2 shRNA pLKO and scrambled shRNA pLKO vectors, respectively. (D) Proliferation rates of H1299 LeGO-Pirh2, control cell line H1299 LeGO, and H1299 Pirh2 shRNA cells. H1299 cells with scrambled shRNA were used as control. The data are shown as cell index graphs.

To generate H1299 cells with different status of Pirh2 we stably transduced these cells with lentiviral (LeGO and pLKO) vectors that express Pirh2 cDNA or specific shRNA against this gene, respectively. Cells with empty LeGO and pLKO expressing scrambled shRNA were used as appropriate controls. The efficiency of transduction was verified by FACs analysis as shown in Figure 1 A. To evaluate the levels of Pirh2 either overexpression or down-regulation mediated by LeGO-Pirh2 and pLKO Pirh2 shRNA vectors, respectively, we used western blotting (Figure 1B). As most of E3 ubiquitin ligases Pirh2 undergoes auto-ubiquitination followed by proteasomal degradation. Therefore, to enhance the Pirh2 western blot signal we treated stably transduced cells with the proteasome inhibitor, MG132. As shown in Figure 1B samples with stable overexpression of Pirh2 in H1299 cells was readily detected by Pirh2-specific antibody. MG132 treatment (right panel) further augmented the signal (Figure 1B). We also evaluated the efficacy of shRNA-mediated knockdown of Pirh2 by comparing Pirh2 western blot signals in control cells (scrambled shRNA) and cells with attenuated expression of Pirh2 (Pirh2 shRNA) (Figure 1C). We found that stable expression of Pirh2 shRNA construct effectively attenuated endogenous expression of Pirh2.

In order to measure the proliferation rate of H1299 cells with different status of Pirh2 we performed real-time monitoring of cell growth using the xCELLigence system (Figure 1D). This system (used hereafter) allows estimating cell index in real time – the parameter based on impedance measurement and reflecting the number of cells attached to the surface of the experimental chamber. Using this instrument, we showed that Pirh2 overexpression (LeGO-Pirh2) promoted cell proliferation while silencing of Pirh2 by shRNA (shRNA-Pirh2) attenuated the proliferative potential of H1299 cells (Figure 1D). Importantly, appropriate controls (LeGO and scrambled cells) exhibited very similar proliferation rates strongly suggesting that these effects were specific to Pirh2 and not to transduction manipulations.

### Pirh2 enhances the migratory potential of H1299 cells

**Figure 2:**
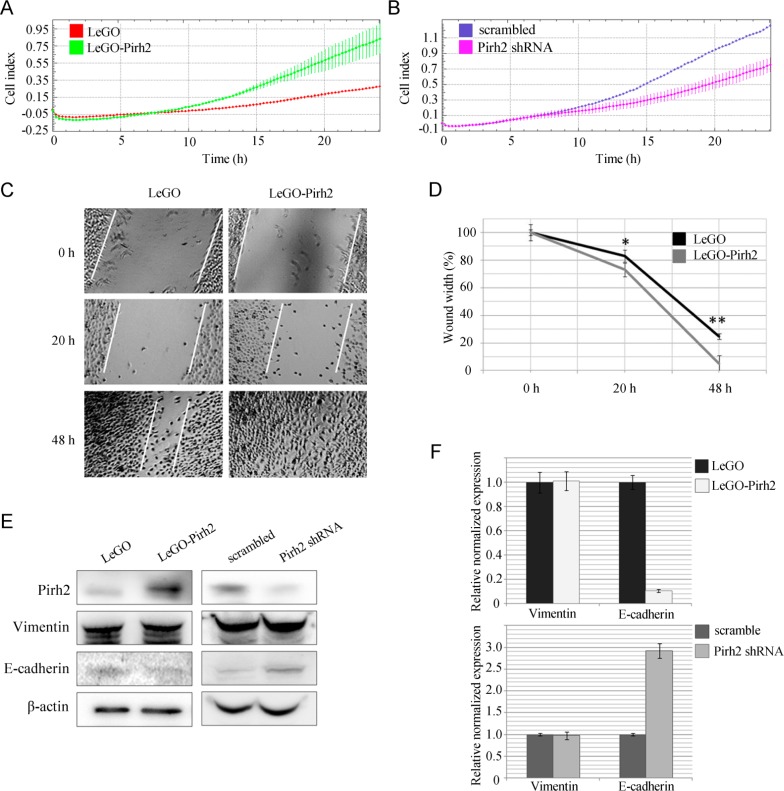
Pirh2 enhances the migratory potential of H1299 cells (A) The migratory activity of H1299 LeGO-Pirh2 cells and control H1299 LeGO cells shown as cell index graphs. (B) The migratory activity of H1299 Pirh2 shRNA cells and control H1299 scrambled shRNA cells shown as cell index graphs. (C) The wound-healing assay using LeGO-Pirh2- and control LeGO-expressing H1299 cells. (D) Statistical results of wound-healing assay. Error bars indicate ± SD. Student's *t*-test was performed for Pirh2-LEGO H1299 cells and their appropriate H1299 LEGO controls. * P<0.05; ** P<0.01. Protein (E) and mRNA (F) expression levels of vimentin and E-cadherin in LeGO-Pirh2, control LeGO, Pirh2 shRNA and control scrambled shRNA H1299 cells.

Next, we wanted to examine the effect of Pirh2 on migratory potential of H1299 cells. To address this question, we used the CIM-plate device of xCELLigence system. CIM-plate consists of two chambers separated by microporous membrane (pore size is 8 μm) attached to microelectrodes. In this case, cell index calculated on the base of impedance measurements reflects the amount of cells migrated through micropores. Our results showed that Pirh2 overexpression enhanced the migration rate compared to control H1299 cells (Figure 2A). On the contrary, silencing of Pirh2 by shRNA resulted in attenuation of H1299 cell migration (Figure 2B). The participation of Pirh2 in regulation of the migration ability of H1299 cells was also confirmed by wound-healing assay. We observed a 50% faster wound closure for LeGO-Pirh2-expressing cells when compared to the control LeGO cells (Figure 2C and 2D). These unexpected results prompted us to evaluate the effect of Pirh2 on the expression of established markers of EMT, vimentin and E-cadherin, which also regulate the migratory potential of cells (Figure 2E and 2D). According to our results, the protein level of E-cadherin is reduced in Pirh2-overexpressing H1299 cells. Accordingly, E-cadherin was increased in Pirh2-deficient H1299 cells. Further, we showed that the effect of Pirh2 on E-cadherin occurs on the transcriptional level since mRNA expression of *CDH1* was attenuated 5-fold upon overexpression of Pirh2 (Figure 2D, upper). In sharp contrast, the level of *CDH1* was increased 3-fold in the cells expressing Pirh2 shRNA compared to the cells with scrambled shRNA (Figure 2D, lower). Importantly, mRNA and protein expression levels of vimentin were unchanged, which argues that Pirh2 specifically affected E-cadherin.

### Pirh2 affects the resistance of H1299 cells to doxorubicin

**Figure 3:**
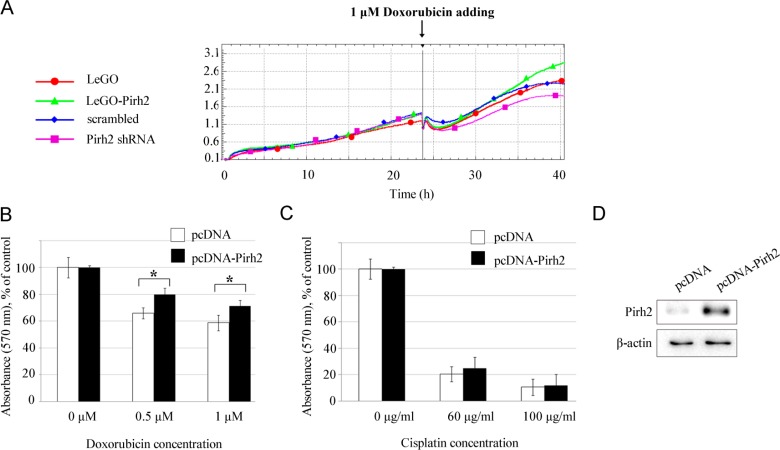
Pirh2 affects the resistance of H1299 cells to doxorubicin (A) Cell index graphs obtained for H1299 cells stably expressing LeGO-Pirh2, control LeGO, Pirh2 shRNA, and scrambled shRNA, respectively, which were exposed to 1 μM doxorubicin. Viability of H1299 cells transfected with the pcDNA-Pirh2 and control pcDNA plasmids after 24 h treatment with doxorubicin (0,5 μM and 1 μM) (B) or cisplatin (60 μg/ml and 100 μg/ml) (C) assessed by MTT assay. Error bars indicate ± SD within quadruplicate. Student's *t*-test was performed for Pirh2 overexpressing samples and their appropriate controls. *P<0.05. (D) Western blot analysis of Pirh2 protein levels in H1299 cells after transfection with pcDNA-Pirh2 or control pcDNA plasmids.

Finally, we assessed the impact of Pirh2 on drug resistance of H1299 cells, which is an important characteristic of curability and aggressiveness of cancer cells. Figure 3 shows the data of real-time proliferation after the treatment with doxorubicin of cell lines with overexpressed (LeGO-Pirh2) and silenced expression of Pirh2 (Pirh2 shRNA), and their controls (LeGO and scrambled, respectively). We observed that Pirh2 overexpression maintained cell proliferation while silencing of Pirh2 significantly reduced the proliferative potential of H1299 cells by 16 hours of treatment (Figure 3A). To confirm these results, we employed MTT assay on H1299 cells transfected with pcDNA or pcDNA-Pirh2 vectors (Figure 3D) treated with doxorubicin or cisplatin. We found that Pirh2 overexpression augmented the viability of H1299 cells treated with doxorubicin but did not affect their resistance to cisplatin (Figure 3B and 3C). These results suggest that Pirh2 selectively benefit cancer cells to resist genotoxic drugs that cause double strand DNA breaks, e.g. doxorubicin, but not the ones that induce cross-linking of DNA strands.

**Figure 4:**
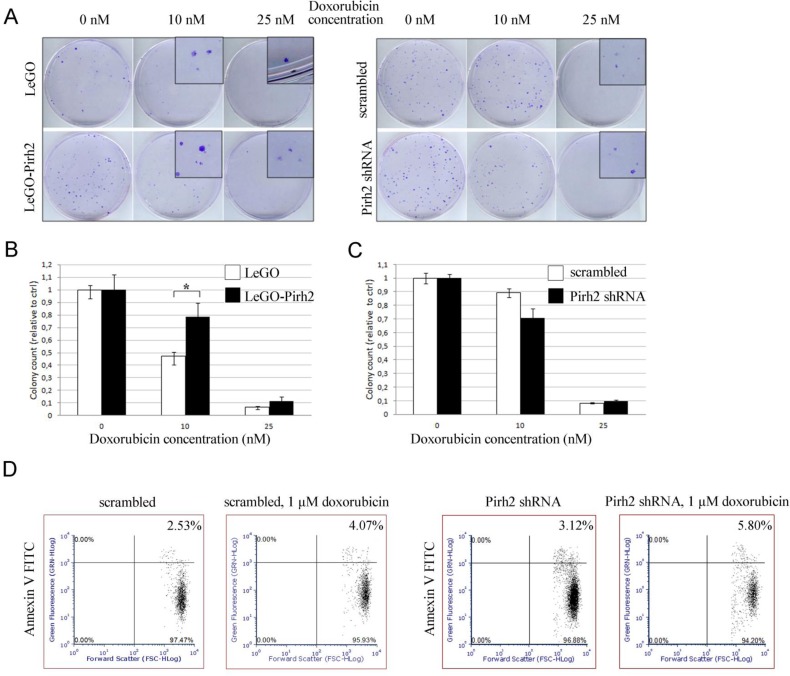
Effect of Pirh2 on colony formation and apoptosis of H1299 cells treated with doxorubicin (A) Results of colony formation assay obtained for H1299 cells stably expressing LeGO-Pirh2, control LeGO, Pirh2 shRNA, and scrambled shRNA, both untreated and treated with 10 nM and 25 nM doxorubicine for 3 days. (B) Statistical analysis of the relative colonies numbers for LeGO-Pirh2 versus LeGO control and (C) Pirh2 shRNA versus scrambled shRNA was carried out. Error bars indicate ± SEM within triplicate, *P<0.05 (Student's *t*-test). (D) The apoptosis rate of Pirh2 shRNA, and scrambled shRNA H1299 cells untreated and treated with 1 μM doxorubicin for 16 hours was determined by Annexin V-FACS analysis.

To substantiate our MTT results we also assessed the effect of Pirh2 on sensitivity of H1299 cells to doxorubicin by colony formation assay. To this end, we compared pair-wise the numbers of colonies formed by control LeGO cells and cells overexpressing Pirh2 (LeGO-Pirh2), and cells expressing scrambled shRNA against H1299 cells with attenuated expression of Pirh2 (Pirh2 shRNA) (Figure 4A, B, and C). Overexpression of Pirh2 increased the number of colonies both in untreated and treated with doxorubicin cells (Figure 4A, left panel and 4B). On the contrary, attenuation of Pirh2 elicited an opposite effect (Figure 4A, right panel and 4C) by increasing the sensitivity of cells to doxorubicin compared to scramble shRNA-expressing cells. It should be noted that the difference in cytotoxicity between the cell line variants became noticeable only at 10 nM concentration of doxorubicin. Yet, at the concentration of 25 nM of doxorubicin the colony formation was almost completely suppressed in all the observed cell lines. Based on these results we estimated the ID50 concentration of doxorubicin for colony formation of H1299 LeGO cells ∼10-11 nM and H1299 LeGO-Pirh2 cells ∼ 15-16 nM. These results strongly suggest that Pirh2 promotes resistance of cancer cells to doxorubicin.

Furthermore, we determined the effect of Pirh2 on apoptosis in response to doxorubicin treatment. Analysis of Annexin V staining of H1299 cells with different levels of Pirh2 expression (scrambled versus shRNA-Pirh2) by FACS revealed the increased level of apoptosis in Pirh2-deficient cells compared with control (Figure 4D) both before and after doxorubicin treatment. These results further confirm the contribution of Pirh2 to proliferation and resistance of H1299 cells to doxorubicin.

### Pirh2 enhances c-Myc expression, which correlates with poor survival of lung cancer patients

**Figure 5:**
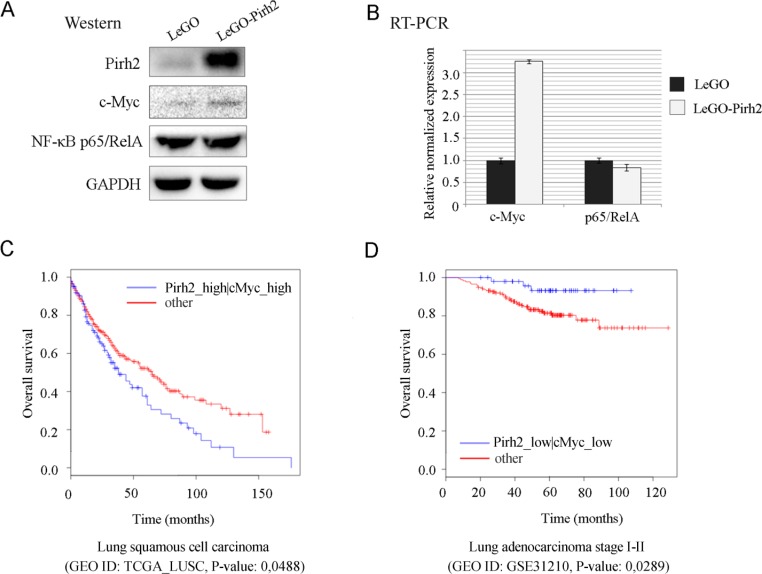
Pirh2 augments c-Myc expression and correlates with poor survival of lung cancer patients Protein (A) and mRNA (B) expression levels of c-Myc and NF-κB p65/RelA in LeGO-Pirh2 and control LeGO H1299 cells. The bioinformatics analysis of synergistic effect of Pirh2 and c-Myc co-expression on (C) lung squamous cell carcinoma patients survival (GEO dataset ID: TCGA_ LUSC), and (D) lung adenocarcinoma stage I-II patients survival (GEO dataset ID: GSE31210), p-values are indicated.

To elucidate the potential molecular mechanism that underlies the tumorigenic properties of Pirh2 we examined expression levels of two the most critical master-regulators of proliferation and cell survival, NF-κB and c-Myc. Control H1299 cells and cells stably expressing Pirh2 were subjected to western blotting analysis using NF-κB p65/RelA- and c-Myc-specific antibodies (Figure 5A). Surprisingly, we found that while p65/RelA expression levels were the same in control and LeGO-Pirh2 cells, the level of c-Myc protein was increased in cells overexpressing Pirh2 (Figure 5A). To get further insights into this observation we measured transcription of both p65/RelA and c-Myc genes as a function of Pirh2 overexpression. Interestingly, we noticed over 3-fold increase of c-Myc transcription in the presence of ectopic Pirh2 compared to control cells, whereas RelA levels were comparable in both types of cells (Figure 5B).

To corroborate these results in the biological setting we carried out the bioinformatics analysis to establish a correlation between the Pirh2 and c-Myc co-expression and survival of lung cancer patients using the Syntarget software as described previously [32]. Interestingly, the elevated expression of *RCHY1* by itself correlated with better survival of patients with different tumors ([24] and data not shown). In contrast, as shown at Figure 5C, high levels of co-expression of the Pirh2-coding *RCHY1* gene and c-Myc correlated significantly with poor survival outcome (Figure 5C). On the contrary, when the expression of both RCHY1 and c-Myc genes was low, survival outcomes of patients was significantly improved (Figure 5D). Thus, it can be hypothesized that one of the potential mechanisms by which Pirh2 increases proliferation, the migratory ability and drug resistance of H1299 cells may involve the augmented expression of c-Myc, which is known to regulate the aforementioned processes.

## DISCUSSION

Recent studies have brought attention to Pirh2 as an E3 ubiquitin ligase that regulates cellular homeostasis upon genotoxic stress both in the p53-dependent and p53-independent cellular context. Several important targets of Pirh2 playing key roles in apoptosis, cell cycle progression and DNA repair have been identified. These include the p53 family proteins (p53, p63 and p73), Chk2, p27^Kip1^ and Polη [12, 14-19]. However, the role of Pirh2 in tumorigenesis is still controversial as expression of human Pirh2 in lung, ovarian, and breast cancers correlates with decreased patients' survival [24]. Both experimental and bioinformatics data presented in this study strongly suggest that Pirh2 functions as an oncogene in lung non-small cell carcinomas. Forced expression of Pirh2 increases the proliferative potential, drug resistance and migration ability of H1299 cells, which is in agreement with experimental data of other researchers. For example, Yuan et al. (2008) reported that Pirh2 silencing by siRNA resulted in reduction of cell proliferation and increased apoptosis in p53-positive A549 lung adenocarcinoma cells [26]. The authors proposed that these effects were caused by Pirh2-mediated degradation of p27^Kip1^ cell cycle inhibitor. However, this speculation requires experimental confirmation. Furthermore, knockdown of Pirh2 by siRNA was reported to suppress the colony formation potential of H1299 cells [14]. Jung and colleagues also demonstrated that Pirh2 overexpression deceased the number of cells in sub-G1 stage after doxorubicin treatment indicating that Pirh2 attenuated the level of apoptosis [14].

Increased resistance to pharmacological treatments is an important feature of aggressive tumors. In this respect, our results showed that ectopically expressed Pirh2 conferred selective resistance to doxorubicin. Doxorubicin induces double-strand breaks in DNA by inhibiting the ligase activity of DNA topoisomerase II. On the contrary, cisplatin mostly makes intra-strand cross-links of purine bases thereby activating nucleotide excision repair (NER) [27]. Based on these finding we speculate that Pirh2 specifically promotes double-strand breaks DNA repair by yet not fully understood mechanism. In this respect, it should be noted that our biochemical screening for novel Pirh2-interacting proteins revealed several new partners of Pirh2 involved in double-strand breaks DNA repair by both homologous recombination and non-homologous end joining (data not published).

Finally, we showed that Pirh2 promoted migratory potential of H1299 cells. Cellular migration and invasion are critical for the formation of metastases. This process is preceded by epithelial-to-mesenchymal transition, which converts epithelial cells into mesenchymal by silencing E-cadherin and augmenting expression of vimentin. Our results demonstrated that ectopic expression of Pirh2 further attenuated cellular levels of E-cadherin, the protein responsible for tight junctions between cells, while keeping the level of vimentin intact. This decrease of E-cadherin expression was paralleled by increased migration of Pirh2-overexpressing cells. It is likely, that in addition to E-cadherin, Pirh2 down-regulates several other gene products that inhibit migration. However, this interesting possibility needs to be tested experimentally.

To get insights into the possible mechanisms by which Pirh2 facilitates proliferation and migration of H1299 cells, we focused on two major regulators of cell proliferation and migration: c-Myc and NF-κB [28-31]. While Pirh2 did not affect NF-κB expression, unexpectedly, we found that Pirh2 stabilized c-Myc (Figure 5A). Our results contrasted the previously published ones on Pirh2-mediated ubiquitin-dependent degradation of the c-Myc protein [24]. In addition, we demonstrated that ectopically expressed Pirh2 induced c-Myc transcription (Figure 5B). To corroborate these seemingly contradictory results one can assume that Pirh2 affects c-Myc expression indirectly and this effect strongly depends on a particular cellular context. As a possible explanation of Pirh2-mediated regulation of c-Myc, it can be speculated that it is mediated via the degradation of p73, which is known to repress c-Myc expression and yet it is an established target of Pirh2 [14, 32]. Therefore, attenuation of p73 by Pirh2 should augment the c-Myc expression. This interesting possibility will need experimental validation in the future. It should also be noted that the c-Myc protein was reported to suppress E-cadherin by activating E-cadherin-specific microRNA miR-9 that mediates attenuation of E-cadherin expression [33]. This fact is consistent with our results showing that Pirh2-mediated up-regulation of c-Myc was concomitant with E-cadherin repression (Figure 5A and 2E).

In summary, this study provides several lines of evidence that Pirh2 plays the oncogenic role in p53-negative human non-small cell lung carcinoma cells by enhancing their proliferation, migration, and resistance to doxorubicin. Thus, our findings suggest that Pirh2 can be considered as a potential target for the development of anticancer therapies aimed at p53-negative tumors.

## MATERIALS AND METHODS

### Plasmids

The pcDNA-Pirh2 construct was obtained from S. Benchimol. For generating stable cell lines either overexpressing Pirh2 or a knockdown of Pirh2 lentiviral vectors LeGO-iG2 [34] and pLKO.1-TRC [35] were used, respectively. The following Pirhe-speicific shRNA oligonucleotides were annealed prior to cloning into the pLKO.1-TRC vector digested with Age1 and EcoR1 enzymes: sense5′-CCGGAATGTAACTTATGCCTAGCT ACTCGAGTAGCTAGGCATAAGTTACATTTTTTTG-3′ and antisense 5′-AATTCAAAAAAATGTAACTTATGCC TAGCTACTCGAGTAGCTAGGCATAAGTTACATT-3′. Scrambled shRNA was used as a control. All obtained expression constructs were verified by sequencing. The lentiviral packaging plasmid psPAX2 and the envelope plasmid pMD2.G were a gift from Didier Trono (Addgene plasmids # 12260 and #12259).

### Manipulations with cells

NCI-H1299 ATCC human non-small cell lung carcinoma cell line was cultured under standard conditions in RPMI 1640 medium (Thermo Fisher Scientific, CA, USA) supplemented with 10% fetal bovine serum (FBS, Lonza, MD, USA), penicillin/streptomycin and 2 mM L-glutamine. H1299 cells were transfected using X-tremeGENE HP reagent (Roche, Switzerland) according to the manufacturer's instructions. Stable cell lines were obtained using multiple rounds of transduction by lentiviral particles. Briefly, HEK293-T cells seeded on 10 cm plate 24 h before were transfected with plasmid mix (15 μg expression vector, 9.35 μg psPAX2 and 5.32 μg pMD2.G) by modified calcium phosphate transfection method [36]. The medium was changed 16 h after transfection and then lentiviral particles were collected every 24 h. To concentrate lentiviral particles an ultracentrifugation procedure through sucrose cushion (2 h 72.000 g) was performed. The lentivirus titer was estimated on HEK293-T by limiting dilution assay. For the transduction procedure, 50,000 cells per well in full RPMI 1640 medium were seeded onto the 24-well plate. After 4 h Polybrene was added to each well to the final concentration 8 μg/ml. Lentiviral particles were added to the cells with subsequent incubation for 24 h. Four days after transduction the percentages of LeGO-iG2-transduced cells were assessed by GFP expression using flow cytometer Guava EasyCyte 8 (EMD Millipore, MA, USA). The cells successfully transduced with pLKO.1-TRC were subjected to puromycin selection (0.5 μg/ml) for 7 days. Pirh2 expression in all obtained cell lines was checked by Western blot analysis.

### Real-time cell proliferation and cell migration assays

The tests were performed using xCELLigence system (ACEA Biosciences, CA, USA). For cell proliferation assays 2×10^4^ cells were seeded in each well of E-plate 16 (ACEA Biosciences, CA, USA) in RPMI 1640 medium. Cell index was registered every 10 minutes. For cell migration assays 3×10^4^ cells were seeded in each well of CIM-plate 16 (ACEA Biosciences, CA, USA) in full RPMI 1640 medium. Cell index was registered every 15 minutes.

### Wound healing (scratch) assay

Cells were seeded in 12-well plates at 80% confluence in full RPMI 1640 medium. The next day linear scratches of the monolayer cell culture were created by 1000 μl pipette tips. The scratch closure was observed and photographed at 0 h, 20 h and 48 h using TS100 inverted light microscope (Nikon, Japan).

### Colony formation assay

The assay was performed essentially as described previously in [37]. Briefly, 500 cells were seeded at 10 cm culture plates in full RPMI 1640 medium for 24 h and then treated with 10 nM and 25 nM doxorubicin for 3 days. Then doxorubicin-containing medium was replaced with full RPMI 1640 medium and incubated for additional 7 days for colony formation. The colonies were fixed and stained for 10 minutes with fixing/staining solution containing 0.05% crystal violet, 1% formaldehyde, 1% methanol buffered with PBS. Following washes and drying the colonies were scored and analyzed manually. The experiments were performed in triplicates.

### MTT assay

24 h after transfection cells were seeded into 96-well plates at 3.000 cells per well in full RPMI 1640 medium for 24 h with subsequent 24 h treatment with doxorubicin (0.5 μM and 1 μM) and cisplatin (60 μg/ml and 100 μg/ml). Then 20 μl 5 mg/ml of Triazolyl Blue (MTT) solution was added to each well for 4 h at 37°C. After removing MTT containing medium, 150 μl isopropyl alcohol (acidified with 40 mM HCl) was added to dissolve MTT-formazan salt. The absorbance at 570 nm and 630 nm was measured using Pikon multiplate reader (Analytica, Russia).

### Annexin V staining

For Annexin V staining the FITC Annexin V Apoptosis Detection Kit (BD Pharmingen, CA, USA) was used according to the manufacture's instructions. Briefly, cells were washed with cold PBS and resuspended in the binding buffer provided by the manufacturer. 5 μl of FITC Annexin V was added to 100 μl of cell suspension and incubated for 20 min. Analysis was performed using Guava EasyCyte 8 flow cytometer (Guava Technologies, Millipore, Billerica, MA, USA) (EMD Millipore, MA, USA).

### Quantitative PCR

The standard procedure of total RNA extraction was performed using TRI Reagent (Sigma-Aldrich, MO, USA) according to the manufacturer's instructions. To eliminate traces of DNA total RNA was treated with DNase I (Thermo Fisher Scientific, CA, USA) according to the manufacturer's instructions and stopped with a standard phenol/chloroform extraction. The reverse transcription reaction with 2 μg of total RNA was performed using RevertAid RT kit (Thermo Fisher Scientific, CA, USA). Quantitative Real Time PCR was carried out using SsoFast EvaGreen Master Mix (BioRad, CA, USA) in the BioRad CFX-96 real time system (BioRad, CA, USA). Relative expression was calculated using the ΔΔCt method. Sample Ct values were normalized to GAPDH. The oligonucleotides used for qPCR were as follows: GAPDH sense 5′-GAGGTCAATGAAGGGGTCAT-3′ and antisense 5′-AGTCAACGGATTTGGTCGTA-3′; Vimentin sense 5′-TGTCCAAATCGATGTGGATGTTTC-3′ and antisense 5′-TTGTACCATTCTTCTGCCTCCTG; E-cadherin sense 5′-CTTCTGCTGATCCTGTCTGATG-3′ and antisense 5′-TGCTGTGAAGGGAGATGTATTG-3′; c-Myc sense 5′-CTCCTCCTCGTCGCAGTAGA-3′ and antisense 5′-GCTGCTTAGACGCTGGATTT-3′; RelA/p65 sense 5′-CGAATGGCTCGTCTGTAGTG-3′ and antisense 5′-TGGTGGTATCTGTGCTCCTC-3′. All amplifications were performed in triplicates.

### Western blotting

For western blot analysis whole-cell extracts were prepared. The primary antibodies against the analyzed proteins were used as follows: Pirh2 (1:1,000, EPR14980, Abcam, CA, USA); GAPDH (1:2,000, ab9484, Abcam, CA, USA); β-actin (1:5,000, A3854, Sigma-Aldrich, MO, USA); NF-κB p65 (1:1,000, sc-8008, Santa Cruz, CA, USA); c-Myc (1:300, 9E10, Santa Cruz, CA, USA); Vimentin (1:5,000, RV202, BD Biosciences, CA, USA); E-cadherin (1:1,000, 36/E, BD Biosciences, CA, USA). Secondary antibodies used were anti-mouse and anti-rabbit (1:10,000; Sigma-Aldrich, MO, USA).

### Bioinformatics

Correlations of expression levels of *RCHY1* (Pirh2) alone and in combination with *myc* (c-Myc) with the survival rates of lung cancer patients were calculated by algorythms described by Amelio et al. [38]. Individual contributions of *RCHY1* and *MYC* genes were examined as described in Antonov et al. [39, 40] using publically available Gene Expression Omnibus (GEO) microarray data.

### Statistical analysis

Data are shown as mean ± standard deviation (SD) or standard error of the mean (SEM) of at least three replicates. Statistical significance was analyzed using Student's t-test. P<0.05 was considered significant. P<0.05 is denoted as *, P<0.01 as**.

## ABBREVIATIONS

ATCC: American Type Culture Collection
Chk2: checkpoint kinase 2
EMT: epithelial-to-mesenchymal transition
GFP: green fluorescent protein
HCC: hepatocellular carcimoma
TNM: Tumor-Node-Metastasis classification of malignant tumours
HNSCC: head and neck squamous cell carcinoma
NFκB: nuclear factor-kappa B
Polη: DNA polymerase eta
RCHY1: RING finger and CHY zinc finger domain-containing protein 1.
